# Network pharmacology and molecular docking study on the treatment of polycystic ovary syndrome with angelica sinensis- radix rehmanniae drug pair

**DOI:** 10.1097/MD.0000000000036118

**Published:** 2023-11-17

**Authors:** Xinghua Li, Ihsan Ullah, Chunxia Hou, Yuqiang Liu, Keyuan Xiao

**Affiliations:** a Changzhi People’s Hospital Affiliated to Changzhi Medical College, Changzhi, China; b National Chinmedomics Research Center, Heilongjiang University of Chinese Medicine, Harbin, China.

**Keywords:** *angelica sinensis*, molecular docking, network pharmacological, polycystic ovary syndrome, *radix rehmanniae*

## Abstract

This study aimed to investigate the *angelica sinensis* - *radix rehmanniae* (AR) role in polycystic ovary syndrome (PCOS), employing network pharmacology and molecular docking techniques for active ingredient, targets, and pathway prediction. AR active components were obtained through TCMSP platform and literature search. The related targets of AR and PCOS were obtained through the disease and Swiss Target Prediction databases. An “active ingredient-target” network map was constructed using Cytoscape software, and gene ontology and Kyoto encyclopedia of genes and genomes enrichment analysis was conducted through Hiplot. Finally, Auto Dock Tools software was used to conduct molecular docking between active ingredients and core targets. The main bioactive ingredients of AR in the treatment of PCOS are acteoside, baicalin, caffeic acid, cistanoside F, geniposide, etc. These ingredients involve 10 core targets, such as SRC, HSP90AA1, STAT3, MAPK1, and JUN. The effect of AR on anti-PCOS mainly involves the AGE-RAGE signaling pathway, Relaxin signaling pathway, TNF signaling pathway, and ErbB signaling pathway. Molecular docking results showed that the main active components and key targets of AR could be stably combined. AR can improve hyperandrogen status, regulate glucose homeostasis, and correct lipid metabolism and other physiological processes through multi-component, multi-target, and multi-pathway. Thus, it could play a significant role in PCOS treatment. The results of our study provide a scientific foundation for basic research and clinical applications of AR for the treatment of PCOS.

## 1. Introduction

Polycystic ovary syndrome (PCOS) is the most common gynecological endocrine disorder among women of reproductive age,^[1]^ characterized by irregular menstruation, hyperandrogenism, and polycystic ovaries.^[2]^ PCOS is one of the major causes of infertility in women of reproductive age,^[3]^ accounting for 70% of ovulation-disordered infertility,^[4]^ and its prevalence in women of reproductive age is 6% to 10%.^[5]^ The etiology of polycystic ovary syndrome is complex, mainlycaused by genetic and environmental factors.^[6]^ In recent years, the incidence of PCOS has increased significantly,^[7]^ and it is often accompanied by complications such as diabetes, abnormal uterine bleeding, infertility, and high androgen expression,^[8]^ which has brought a heavy economic burden to patients and society. Therefore, PCOS treatment is a hot topic in current medical research. Currently, the treatment drugs for PCOS mainly include ovulation-stimulating drugs, insulin resistance-improving drugs, and androgen-lowering drugs. Still, these drugs are limited to symptomatic treatment, easy to relapse after drug withdrawal, require repeated treatment, and are prone to ovarian hyperstimulation syndrome, premature ovarian failure, lactic acid poisoning, increased cardiovascular burden, and other side effects.^[9]^ New treatments are urgently needed. Traditional Chinese medicines (TCM) have the advantages of having fewer side effects and acting on multiple targets and pathways. Several clinical studies have demonstrated that TCM has achieved significant clinical effectiveness in restoring the menstrual cycle, regulating sex hormone levels and glucose-lipid metabolism, as well as increasing ovulation rates and conception rates.,^[10]^ so the study of TCM used in treating polycystic ovary syndrome has a broad application prospect.

Herbal pairs, the basic constituent units of herbal formulas, have special clinical significance in Chinese-medicine and have gradually become a hotspot of modern research.^[11]^ Compared with a single drug, herbal pairs can act synergistically to promote the treatment of various diseases. Modern research shows that *Angelica sinensis* has antitumour, liver protection, anti-inflammatory, hypoglycemic, immune regulation, cardiovascular and cerebral vascular system protection, etc,^[12]^ and *Radix Rehmanniae* has the effect of regulating blood lipids and blood glucose, antiaging, antitumour, antibacterial, and protecting gastric mucosa, etc.^[13]^ The pair “*angelica sinensis* - *radix rehmanniae* (AR)” was first recorded in “Taiping Huimin Hejiaobu Fang”, which is a commonly used pair in TCM. *Angelica sinensis* and *Radix Rehmanniae* are 2 Chinese medicines with the highest frequency of use and the most frequent combinations of medicines in treating PCOS. However, their mechanism of action in PCOS treatment is still poorly understood.

The progress of network pharmacology relies on a more profound grasp of how molecules and proteins interact with each other. There are great opportunities to gain a better understanding of diseases, how they develop TCM syndromes, and their treatment mechanisms. It has been applied to the field of TCM, including drug target discovery, efficacy evaluation, and mechanism research. A variety of applications have been made to TCM, including the discovery of drug targets, evaluation of efficacy, and investigation of mechanisms. In order to better understand the mechanism of action of TCM, network pharmacology is an effective method. In this paper, we explored the active ingredients of AR using network pharmacology methods and predicted their targets and signaling pathways related to PCOS, built a disease-drug-ingredient-target network, and analyzed the targets and signaling pathways that could be involved in the treatment of PCOS by AR.

## 2. Materials and methods

### 2.1. AR active component screening and target prediction

In this study, the compound compositions of *Angelica sinensis* and *Radix Rehmanniae* were searched through the TCMSP database (https://tcmsp-e.com/tcmsp.php).^[14]^ Based on pharmacokinetic principles and platform recommendations, the chemical components were obtained as the active ingredients of the 2 Chinese medicines. In the search results, *Angelica sinensis* and *Radix Rehmanniae* had oral bioavailability of ≥ 30% and drug-like properties of ≥ 0.18. Also, based on a literature search, ingredients with pharmacodynamic effects were included as active ingredients. A database containing active ingredient information has been integrated with PubChem (https://pubchem.ncbi.nlm.nih.gov/).^[15]^ Download the Smiles information for each component and enter it into the Swiss Target Prediction database (http://www.Swisstargetprediction.ch/).^[16]^ The target proteins corresponding to each component were obtained by setting the attribute as “human” and filtering out targets with “Probability” > 0 as potential targets.

### 2.2. Screening of AR targets for the treatment of PCOS and its network construction

The Genecard database (https://www.genecards.org/)^[17]^ and OMIM database (https://www.ncbi.nlm.nih.gov/omim/)^[18]^ were searched for disease targets related to PCOS using the keywords “polycystic ovary syndrome”. Obtaining PCOS-related targets was achieved by combining the results of the 2 databases and de-weighting them.

The drug target and disease target were taken to intersect to obtain the common target, which is the target of AR drug pair for PCOS treatment. In order to construct the drug-active ingredient therapeutic target network, Cytoscape v3.8.0 (www.cytoscape.org/) was utilized.^[19]^ Compounds with the top 10 Degree values were then selected to screen them as the key compounds, considering them to have an essential role in the treatment of PCOS by AR.

### 2.3. Screening for core targets based on protein interaction networks

A String database (https://string-db.org/),^[20]^ was used to construct protein interactions with therapeutic targets, with a threshold value > 0.900 for filtring nonspecific interactions. Cytoscape 3.8.0 software was employed for visualizing the protein interaction network. The 10 highest-degree targets are considered core targets for subsequent molecular docking.

### 2.4. Gene ontology (GO) enrichment analysis and Kyoto encyclopedia of genes and genomes (KEGG) pathway enrichment analysis

The Hiplot platform (https://hiplot-academic.com/),^[21]^ was utilized for GO and KEGG pathway enrichment analysis. GO enrichment analysis provided results for Biological Processes, Molecular Functions, and Cellular components. We create a Bubble plot for the top 20 entries based on KEGG enrichment analysis with a *P* value < .05.

### 2.5. Molecular docking validation

**Figure 1.**
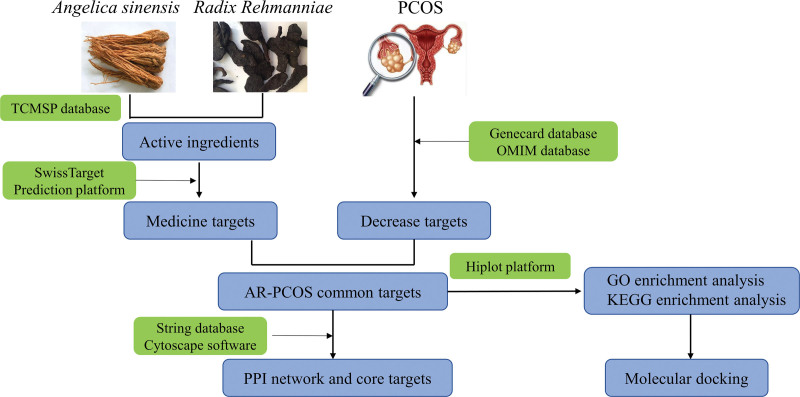
Flow chart of the study.

By employing restricted species “homosapiens,” with an acceptable resolution of < 2.5, and small molecule compounds as reference criteria, we conducted screening of suitable target protein structures in the RCSB database (http://www.rcsb.org).^[22]^ We used a PubChem database to download SDF files of the 3D structures of the active ingredients. The target protein was dehydrogenated using Auto Dock software.^[23]^ The Box size and position were adjusted to make the ligand and receptor into the optimal conformation, and the results were output in “pdbqt” format. We visualized A molecular docking result with PyMol 2.4.0 software.^[24]^ Our flow chart of the study is shown in Figure 1.

## 3. Results

### 3.1. Acquisition of drug-active ingredients and targets

A total of 125 chemical components of *Angelica sinensis* and 76 chemical components of *Radix Rehmanniae* were retrieved, and 14 active components of *Angelica sinensis* and 20 active components of *Radix Rehmanniae* were obtained after screening, among which 2 active components were common to both *Angelica sinensis* and *Radix Rehmanniae* (Table 1). According to the Swiss Target Prediction platform, 421 targets were obtained after removing duplicates, 339 targets were obtained for *Angelica sinensis*, and 215 for *Radix Rehmanniae*.

**Table 1:** TCM Active ingredients.

Chinese-medicine	Chemical-compounds	Formula	Molecular weight (g/mol)
Angelica sinensis, *radix rehmanniae*	Beta-sitosterol	C_29_H_50_O	414.71
Angelica sinensis, *radix rehmanniae*	Stigmasterol	C_29_H_48_O	412.70
Angelica sinensis	Ferulic acid	C_10_H_10_O_4_	194.18
Angelica sinensis	Ligustilide	C_12_H_14_O_2_	190.24
Angelica sinensis	Sedanolide	C_12_H_18_O_2_	194.27
Angelica sinensis	Coniferyl ferulate	C_20_H_20_O_6_	356.37
Angelica sinensis	Butylidene phthalide	C_12_H_12_O_2_	188.22
Angelica sinensis	Chlorogenic acid	C_16_H_18_O_9_	354.31
Angelica sinensis	Senkyunolide I	C_12_H_16_O_4_	224.25
Angelica sinensis	Senkyunolide A	C_12_H_16_O_2_	192.25
Angelica sinensis	Senkyunolide H	C_12_H_16_O_4_	224.25
Angelica sinensis	Butylphthalide	C_12_H_14_O_2_	190.24
Angelica sinensis	Levistolide A	C_24_H_28_O_4_	380.48
Angelica sinensis	Senkyunolide B	C_12_H_12_O_3_	204.22
*Radix rehmanniae*	Catalpol	C_15_H_22_O_10_	362.33
*Radix rehmanniae*	Acteoside	C_29_H_36_O_15_	624.59
*Radix rehmanniae*	Ajugol	C_15_H_24_O_9_	348.35
*Radix rehmanniae*	Cistanoside F	C_21_H_28_O_13_	488.44
*Radix rehmanniae*	Geniposide	C_17_H_24_O_10_	388.37
*Radix rehmanniae*	Rehmannioside A	C_21_H_32_O_15_	524.47
*Radix rehmanniae*	Echinacoside	C_35_H_46_O_20_	786.73
*Radix rehmanniae*	Aucubin	C_15_H_22_O_9_	346.33
*Radix rehmanniae*	Rehmannioside D	C_27_H_42_O_20_	686.61
*Radix rehmanniae*	Reineckiagenin	C_27_H_44_O_5_	448.64
*Radix rehmanniae*	Rehmapicroside	C_16_H_26_O_8_	346.37
*Radix rehmanniae*	Isoacteoside	C_29_H_36_O_15_	624.59
*Radix rehmanniae*	Vanillic acid	C_8_H_8_O_4_	168.15
*Radix rehmanniae*	Caffeic acid	C_9_H_8_O_4_	180.16
*Radix rehmanniae*	Kaempferol-3-O-glucuronide	C_28_H_32_O_16_	624.54
*Radix rehmanniae*	Baicalin	C_21_H_18_O_11_	446.36
*Radix rehmanniae*	3’,4’-Dihydroxyacetophenone	C_8_H_8_O_3_	152.15
*Radix rehmanniae*	5-Hydroxymethylfurfural	C_6_H_6_O_3_	126.11

TCM = Traditional Chinese medicines.

### 3.2. Screening of AR for the treatment of PCOS targets and its network construction

**Figure 2.**
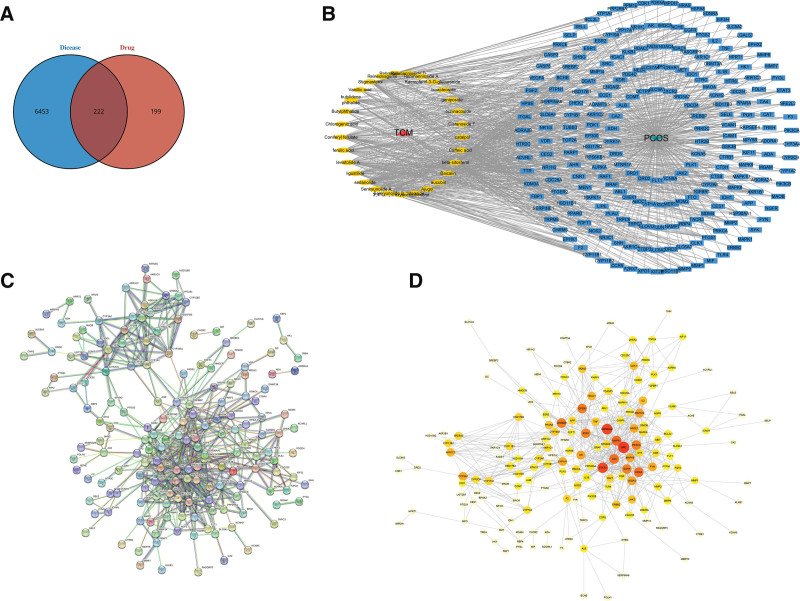
(A) Venn diagram of AR targets and PCOS disease-related targets. (B) Drug-ingredient-target-disease network diagram. (C) PPI network diagram of candidate genes. (D) Topological analysis diagram of the candidate genes. AR = *angelica sinensis – radix rehmanniae*, PCOS = polycystic ovary syndrome.

The Genecard and OMIM databases were searched for disease targets related to PCOS using the keyword “polycystic ovary syndrome”. After de-emphasis, 6675 disease targets were obtained; the intersection of component targets and disease targets was taken to get 222 potential targets of AR for PCOS treatment (Fig. 2A). Cytoscape 3.8.0 software was used to construct a network diagram of “traditional Chinese medicine - active ingredients - therapeutic targets - diseases”. The network contains 253 nodes and 907 edges, with yellow representing the active ingredients and blue representing the targets (Fig. 2B). The protein-protein interaction results of these targets were obtained through the STRING database, and the highest confidence results with a screening threshold higher than 0.9 were screened (Fig. 2C). The network topology analysis of this protein interaction information was performed through the Network Analyzer function of Cytoscape 3.8.0 software, which contained 222 nodes and 610 edges, and colors from light to dark represent Degree values from small to large (Fig. 2D). The targets with the top 10 degree values were screened out as the core targets of AR for the treatment of PCOS, as shown in Table 2. The compounds corresponding to them are acteoside, Baicalin, Caffeic acid, Cistanoside F, geniposide, Isoacteoside, the Kaempferol-3-O-glucuronide, Rehmannioside A, Rehmapicroside, Reineckiagenin, Coniferyl ferulate, ferulic acid, levistolide A, ligustilide sedanolide, Senkyunolide A, Senkyunolide B, can be used as core compounds.

**Table 2:** The core targets of AR for the treatment of PCOS.

Gene	Degree
SRC	39
HSP90AA1	33
STAT3	32
MAPK1	27
JUN	26
HRAS	26
EP300	24
CREBBP	24
PIK3CA	23
EGFR	23

AR = *angelica sinensis - radix rehmanniae*, PCOS = polycystic ovary syndrome.

### 3.3. Enrichment analysis results

**Figure 3.**
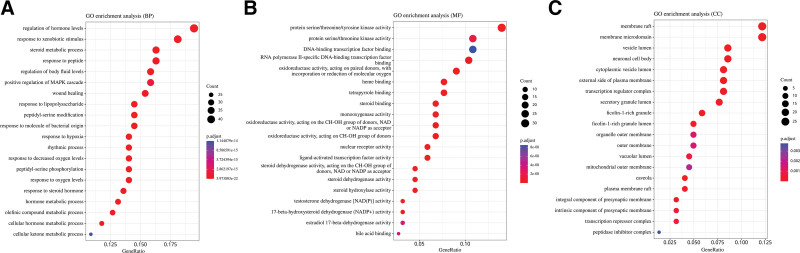
GO enrichment analysis pathway diagram. (A) Enrichment of GO biological process. (B) Enrichment of GO molecular function. (C) Enrichment of GO cellular component. GO = gene ontology.

With *P* value < .05 as the screening condition, a total of 4525 entries were obtained for GO enrichment, and the top-ranked results were visualized in Figure 3. The results of the enrichment analysis showed that these targets could affect biological processes through the regulation of hormone levels, response to xenobiotic stimulus, steroid metabolic process, regulation of body fluid levels, response to lipopolysaccharide, etc; and affect biological processes through membrane raft, membrane microdomain, vesicle lumen, cytoplasmic vesicle lumen, etc; affects cellular composition through protein serine/threonine/tyrosine kinase activity protein serine/ threonine kinase activity, oxidoreductase activity, heme binding.

**Figure 4.**
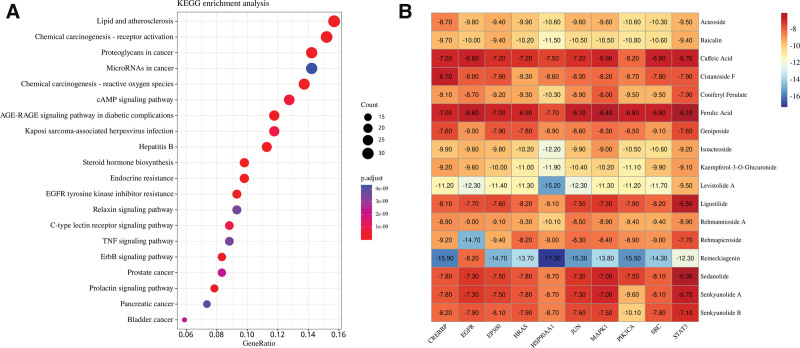
(A) KEGG pathway enrichment diagram. (B) Molecular docking heat map. KEGG = Kyoto encyclopedia of genes and genomes.

A total of 278 KEGG enrichment analysis results were screened with *P* value < .05, highlighting higher-ranking pathways such as 20 AGE-RAGE signaling pathways, Relaxin signaling pathways, TNF signaling pathways, ErbB signaling pathways, and others’’. The angelica-ripened dihuang pair may play a role in these pathways for treating PCOS, and the results are shown in Figure 4A.

### 3.4. Molecular docking results

**Figure 5.**
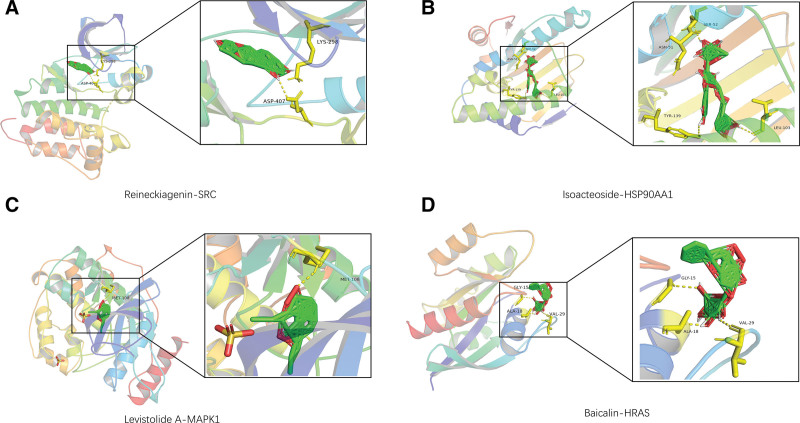
Schematic diagram of molecular docking.

Molecular docking was performed to analyze how the core compounds interact with the core targets. The docking results will then be combined and displayed through heatmaps. PyMol was used to visualize the top-ranked results. A docking score lower than −4.25 kcal/mol indicates some binding activity, while a score lower than −7.0 kcal/mol indicates strong binding activity. The present results showed that the average docking score was −9.14 kJ/mol, among which the results with docking score ≤ −7.0 kJ/mol accounted for 91.17%, which reflected that the compounds in the AR pairs had good binding ability to the core target according to the scoring results. Heatmaps demonstrated all the scoring results, as in Figure 4B. Some molecular docking results were visualized by PyMol 2.4.0, as shown in Figure 5.

## 4. Discussion

PCOS is a polygenic, multifactorial, systemic, inflammatory autoimmune disease. Its pathogenesis is not fully clear.^[25]^ Clinical treatments for PCOS are mainly based on adjusting the menstrual cycle, lowering the level of androgens, and promoting ovulation or surgery, which has more adverse effects and is prone to recurrence.^[26–28]^ It is found that most PCOS patients show increased androgen content, high blood sugar, and lipid metabolism disorders.^[29]^ TCM plays a role in treating PCOS through the advantages of multi-targets and multi-pathways. Clinical studies have shown that the pair of AR and the formula containing AR can significantly improve hyperandrogenism, regulate glucose homeostasis, and correct lipid metabolism abnormality.^[30,31]^ It is therefore necessary to clarify the molecular basis and biological basis of AR against PCOS. This study could provide new directions for further research on the molecular mechanism of PCOS using network pharmacology, a new method for elucidating complex pharmacological problems.

According to the PPI study based on the STRING database and network topology parameters, we further screened 10 core targets: SRC, HSP90AA1, STAT3, MAPK1, JUN, HRAS, EP300, CREBBP, PIK3CA, and EGFR. C-src is a proto-oncogene that regulates cell proliferation, differentiation, and apoptosis. It promotes the activation of primordial follicles through the PI3K, PKC, and MAPK signaling pathways.^[32]^ HSP90AA1, a member of the heat shock family, encodes inducible molecular chaperones that aid in the folding of specific target proteins. HSP90 is mainly found in ovarian granulosa cells, with significantly higher levels in dominant follicles. It plays a role in promoting follicle maturation regulation.^[33]^ STAT3 is an intracellular signaling transcription factor that plays a crucial role in the immune response, cell cycle regulation, and cell survival. Up-regulation of STAT3 expression in porcine ovarian granulosa cells inhibits apoptosis and promotes cell proliferation. It is also involved in inflammation, with increased phosphorylation levels during inflammation. These findings suggest that up-regulating STAT3 expression or inhibiting its phosphorylation could improve ovarian function.^[34]^ MAPK is a crucial signaling pathway linked to androgen production and insulin resistance in PCOS. Its role in the development and maturation of oocytes, as well as the initiation of ovulation, is of utmost importance. Inhibiting the MAPK pathway pharmacologically leads to a decrease in the expression of mitotic cyclin D2 and a reduction in granulocyte proliferation; lower levels of granulocytes indirectly interfere with follicle development and maturation.^[35,36]^ JUN kinase is a significant branch of the mitogen-activated protein kinase signaling pathway. In vivo, high expression of p-c-Jun promotes trophoblast migration and invasion and enhances ovarian function.^[37]^ HRAS belongs to the RAS gene family and acts as an upstream regulator of the RAS/RAF/MEK/ERK/MAPK pathway. In patients with polycystic ovary syndrome, the expression of serum HRAS correlates with metabolic profiles such as body mass index, fasting glucose levels, and fasting insulin levels^[38]^. EGFR, a tyrosine kinase-type receptor, plays a role in crucial reproductive processes like embryo implantation and metaphase. It influences the proliferation and apoptosis of ovarian granulosa cells, thereby inducing oocyte division and maturation. The EGFR gene is predominantly expressed in granulosa cells, and patients with polycystic ovary syndrome exhibit higher levels of EGFR expression in their ovarian tissues, which can impact follicular development.^[38–40]^

Based on the core targets, we obtained 17 key elements that are associated with them. These components can serve as the fundamental active ingredients in the treatment of PCOS using the AR pair. Angelica sinensis contains coniferyl ferulate, ferulic acid, levistolide A, ligustilide, sedanolide, Senkyunolide A, and Senkyunolide B as its active ingredients. Radix Rehmanniae, on the other hand, contains Acteoside, Baicalin, Caffeic Acid, Cistanoside F, Geniposide, Isoacteoside, Kaempferol-3-O-Glucuronide, Rehmannioside A, Rehmapicroside, and Reineckiagenin. Through molecular docking analysis, it was found that Reineckiagenin had the highest docking score with each core target. The study also revealed that Reineckiagenin exhibits estrogen-like activity by binding to estrogen receptors ERα and GPR30. It regulates the hypothalamic-pituitary-ovarian axis, inhibits the secretion of luteinising hormone and follicle-stimulating hormone, and suppresses the production of inflammatory mediators such as iNOS, COX-2, and IL-6. Consequently, it generates anti-inflammatory effects by reducing the concentration of C-reactive protein in the blood plasma.^[41]^

Go enrichment analysis showed that the treatment of PCOS using AR primarily involves the regulation of nutrients, vascular lesions, and oxidative substance metabolism. Various organelles, hormone levels, and steroid metabolism influence this process. It encompasses multiple pathways associated with inflammation and metabolic processes. This indicates that the therapeutic effects of traditional Chinese medicines on this disease are achieved through multiple components, targets, pathways, and biological mechanisms.

Based on the KEGG enrichment analysis, the AGE-RAGE signaling pathway was identified as the most significant therapeutic pathway. This pathway plays a crucial role in mediating insulin resistance and triggering inflammatory responses in polycystic ovaries. Factors like high glucose and obesity lead to non-enzymatic glycosylation of proteins, lipids, and DNA in the body. This, in turn, increases the production of advanced glycation end products, which bind to the RAGE receptor on cell membranes. The binding of advanced glycation end products to RAGE activates the AGE-RAGE signaling pathway, resulting in insulin resistance, inflammation, oxidative stress, and the development of PCOS.^[42,43]^

This study, even though it potentially identifies the targets and signaling pathways of AR in the treatment of PCOS, has certain limitations due to the fact that TCM has great potential in treating PCOS. Network pharmacology uses these newly discovered chemical components of AR as a research topic. TCM is not just a straight collection of chemical-compounds, but both its structure and chemical constituents are transparent. A variety of ingredients in AR can produce different effects depending on their concentrations, contents, and interactions. Moreover, AR pharmacological effects in the treatment of PCOS cannot be fully revealed by the databases used to compile data on targets, components, etc. A scientific hypothesis cannot be divorced from experimental verification - and network pharmacology research is no exception. To verify the accuracy and reliability of the prediction results of the network pharmacology of AR in the treatment of PCOS, it is necessary to combine computational prediction with experimental confirmation and to conduct biological experiments on the prediction results of the network pharmacology of AR. This provides strong evidence to support the development of new Chinese medical treatments for PCOS and other diseases.

## 5. Conclusion

In conclusion, the integration of network pharmacology and molecular docking technology revealed that *Angelica sinensis* and *Radix Rehmanniae* primarily exert their therapeutic effects by targeting multiple molecules such as SRC, HSP90AA1, STAT3, MAPK1, JUN, and others. Through their 17 active ingredients, these herbs regulate lipid metabolism, insulin resistance, and androgen levels to treat PCOS. The main mechanism of action involves the modulation of the AGE-RAGE signaling pathway, which is crucial in PCOS treatment. This study provides insights into the potential pharmacological basis and mechanism of action of AR for PCOS treatment. The network pharmacology approach used here offers a comprehensive analysis of the multi-component, multi-target, functional, and signaling pathway aspects of these herbs in the context of PCOS treatment. This lays the groundwork for future in-depth research and clinical evaluations.

## Author contributions

**Conceptualization:** Keyuan Xiao.

**Data curation:** Ihsan Ullah.

**Funding acquisition:** Keyuan Xiao, Yuqiang Liu.

**Writing – original draft:** Xinghua Li.

**Writing – review & editing:** Chunxia Hou, Yuqiang Liu.

## References

[1] Zhu M Xu Y Li C. Involvement of impaired CD8 (+) mucosal-associated invariant T cells and myeloid-derived suppressor cells in polycystic ovary syndrome. Reprod Biol Endocrinol. 2021;19:175. 34847942 10.1186/s12958-021-00861-7 PMC8630849

[2] Hosseinkhani A Asadi N Pasalar M. Traditional Persian medicine and management of metabolic dysfunction in polycystic ovary syndrome. J Tradit Complement Med. 2018;8:17–23. 29321985 10.1016/j.jtcme.2017.04.006 PMC5755987

[3] Albaghdadi AJH Feeley CA Kan FWK. Low-dose tacrolimus prevents dysregulated peri-conceptional ovarian and systemic immune cellular homeostasis in subjects with PCOS. Sci Rep. 2019;9:6528. 31024070 10.1038/s41598-019-42960-x PMC6484102

[4] Wu H Yu K Yang Z. Associations between TNF-α and interleukin gene polymorphisms with polycystic ovary syndrome risk: a systematic review and meta-analysis. J Assist Reprod Genet. 2015;32:625–34. 25690158 10.1007/s10815-015-0449-7 PMC4380887

[5] Cai M Ni Z Yuan Z. Past and present: a bibliometric study on polycystic ovary syndrome. J Ovarian Res. 2023;16:42. 36803912 10.1186/s13048-022-01072-3 PMC9938353

[6] Sørensen AE Udesen PB Maciag G. Hyperandrogenism and metabolic syndrome are associated with changes in serum-derived microRNAs in women with polycystic ovary syndrome. Front Med (Lausanne). 2019;6:242. 31737638 10.3389/fmed.2019.00242 PMC6839444

[7] Zhou Y Lv L Liu Q. Total flavonoids extracted from Nervilia Fordii function in polycystic ovary syndrome through IL-6 mediated JAK2/STAT3 signaling pathway. Biosci Rep. 2019;39:BSR20181380. 30463907 10.1042/BSR20181380 PMC6328881

[8] Geng Y Sui C Xun Y. MiRNA-99a can regulate proliferation and apoptosis of human granulosa cells via targeting IGF-1R in polycystic ovary syndrome. J Assist Reprod Genet. 2019;36:211–21. 30374732 10.1007/s10815-018-1335-x PMC6420594

[9] Luo ED Jiang HM Chen W. Advancements in lead therapeutic phytochemicals polycystic ovary syndrome: a review. Front Pharmacol. 2022;13:1065243. 36699064 10.3389/fphar.2022.1065243 PMC9868606

[10] Xu W Tang M Wang J. Identification of the active constituents and significant pathways of Cangfu Daotan decoction for the treatment of PCOS based on network pharmacology. Evid Based Complement Alternat Med. 2020;2020:4086864. 32148541 10.1155/2020/4086864 PMC7057008

[11] Liu J Shi JL Guo JY. Anxiolytic-like effect of Suanzaoren-Wuweizi herb-pair and evidence for the involvement of the monoaminergic system in mice based on network pharmacology. BMC Complement Med Ther. 2023;23:7. 36624423 10.1186/s12906-022-03829-1 PMC9830753

[12] Lü J Jiang C Schell TD. Angelica gigas: signature compounds, in Vivo anticancer, analgesic, neuroprotective and other activities, and the clinical translation challenges. Am J Chin Med. 2022;50:1475–527. 35876033 10.1142/S0192415X2250063X

[13] Li M Jiang H Hao Y. A systematic review on botany, processing, application, phytochemistry and pharmacological action of *Radix Rehmnniae*. J Ethnopharmacol. 2022;285:114820. 34767834 10.1016/j.jep.2021.114820

[14] Ru J Li P Wang J. TCMSP: a database of systems pharmacology for drug discovery from herbal medicines. J Cheminform. 2014;6:13. 24735618 10.1186/1758-2946-6-13 PMC4001360

[15] Kim S Thiessen PA Bolton EE. PubChem substance and compound databases. Nucleic Acids Res. 2016;44:D1202–13. 26400175 10.1093/nar/gkv951 PMC4702940

[16] Gfeller D Grosdidier A Wirth M. Swiss target prediction: a web server for target prediction of bioactive small molecules. Nucleic Acids Res. 2014;42:W32–8. 24792161 10.1093/nar/gku293 PMC4086140

[17] Stelzer G Rosen N Plaschkes I. The gene cards suite: from gene data mining to disease genome sequence analyses. Curr Protoc Bioinformatics. 2016;54:1.30.1–1.30.33. 10.1002/cpbi.5 27322403

[18] Amberger JS Hamosh A. Searching online mendelian inheritance in man (OMIM): a knowledgebase of human genes and genetic phenotypes. Curr Protoc Bioinformatics. 2017;58:1.2.1–1.2.12. 10.1002/cpbi.27 PMC5662200 28654725

[19] Kohl M Wiese S Warscheid B. Cytoscape: software for visualization and analysis of biological networks. Methods Mol Biol. 2011;696:291–303. 21063955 10.1007/978-1-60761-987-1_18

[20] Szklarczyk D Gable AL Lyon D. STRING v11: protein-protein association networks with increased coverage, supporting functional discovery in genome-wide experimental datasets. Nucleic Acids Res. 2019;47:D607–13. 30476243 10.1093/nar/gky1131 PMC6323986

[21] Li J Miao B Wang S. Hiplot: a comprehensive and easy-to-use web service for boosting publication-ready biomedical data visualization. Brief Bioinform. 2022;23:bbac261. 35788820 10.1093/bib/bbac261

[22] wwPDB consortium. Protein data bank: the single global archive for 3D macromolecular structure data. Nucleic Acids Res. 2019;47:D520–d528. 30357364 10.1093/nar/gky949 PMC6324056

[23] Morris GM Huey R Lindstrom W. Auto dock4 and Auto dock tools4: automated docking with selective receptor flexibility. J Comput Chem. 2009;30:2785–91. 19399780 10.1002/jcc.21256 PMC2760638

[24] Mooers BHM. Shortcuts for faster image creation in PyMOL. Protein Sci. 2020;29:268–76. 31710740 10.1002/pro.3781 PMC6933860

[25] Escobar-Morreale HF. Polycystic ovary syndrome: definition, aetiology, diagnosis and treatment. Nat Rev Endocrinol. 2018;14:270–84. 29569621 10.1038/nrendo.2018.24

[26] Glintborg D Altinok ML Mumm H. Body composition is improved during 12 months treatment with metformin alone or combined with oral contraceptives compared with treatment with oral contraceptives in polycystic ovary syndrome. J Clin Endocrinol Metab. 2014;99:2584–91. 24742124 10.1210/jc.2014-1135

[27] Teede HJ Misso ML Deeks AA. Assessment and management of polycystic ovary syndrome: summary of an evidence-based guideline. Med J Aust. 2011;195:S65–112. 21929505 10.5694/mja11.10915

[28] Fauser BCJM Tarlatzis BC Rebar RW. Consensus on women’s health aspects of polycystic ovary syndrome (PCOS): the Amsterdam ESHRE/ASRM-Sponsored 3rd PCOS consensus workshop group. Fertil Steril. 2012;97:28–38.e25. 22153789 10.1016/j.fertnstert.2011.09.024

[29] Yu J Zhou Y Ding J. Characteristics and possible mechanisms of metabolic disorder in overweight women with polycystic ovary syndrome. Front Endocrinol (Lausanne). 2022;13:970733. 36714563 10.3389/fendo.2022.970733 PMC9878688

[30] Deng Y Xue W Wang Y-F. Insulin resistance in polycystic ovary syndrome improved by Chinese Medicine Dingkun Pill (定坤丹): a randomized controlled clinical trial. Chin J Integr Med. 2019;25:246–251. 31236888 10.1007/s11655-018-2947-1

[31] Tian J Xu Y Xiong Y. Metabolomics combined with network pharmacology to explore the mechanisms of modified Guishen pill to ameliorate polycystic ovary syndrome. Comput Biol Med. 2022;148:105790. 35839542 10.1016/j.compbiomed.2022.105790

[32] Du XY Huang J Xu LQ. The proto-oncogene C-SRC is involved in primordial follicle activation through the PI3K, PKC, and MAPK signaling pathways. Reprod Biol Endocrinol. 2012;10:58. 22905678 10.1186/1477-7827-10-58 PMC3444437

[33] Driancourt MA Guet P Reynaud K. Presence of an aromatase inhibitor, possibly heat shock protein 90, in dominant follicles of cattle. J Reprod Fertil. 1999;115:45–58. 10341722 10.1530/jrf.0.1150045

[34] Yuan X Zhou X He Y. C/EBPβ promotes STAT3 expression and affects cell apoptosis and proliferation in porcine ovarian granulosa cells. Genes (Basel). 2018;9:295. 29899261 10.3390/genes9060295 PMC6026978

[35] Zhang N Liu X Zhuang L. Berberine decreases insulin resistance in a PCOS rats by improving GLUT4: dual regulation of the PI3K/AKT and MAPK pathways. Regul Toxicol Pharmacol. 2020;110:104544. 31778716 10.1016/j.yrtph.2019.104544

[36] Kandaraki EA Chatzigeorgiou A Papageorgiou E. Advanced glycation end products interfere in luteinizing hormone and follicle stimulating hormone signaling in human granulosa KGN cells. Exp Biol Med (Maywood). 2018;243:29–33. 28914097 10.1177/1535370217731288 PMC5788153

[37] Koski CL Hila S Hoffman GE. Regulation of cytokine-induced neuron death by ovarian hormones: involvement of antiapoptotic protein expression and c-JUN N-terminal kinase-mediated proapoptotic signaling. Endocrinology. 2004;145:95–103. 14512437 10.1210/en.2003-0803

[38] Zheng Q Li Y Zhang D. ANP promotes proliferation and inhibits apoptosis of ovarian granulosa cells by NPRA/PGRMC1/EGFR complex and improves ovary functions of PCOS rats. Cell Death Dis. 2017;8:e3145. 29072679 10.1038/cddis.2017.494 PMC5682660

[39] Large MJ Wetendorf M Lanz RB. The epidermal growth factor receptor critically regulates endometrial function during early pregnancy. PLoS Genet. 2014;10:e1004451. 24945252 10.1371/journal.pgen.1004451 PMC4063709

[40] Yang L Wei Q Li W. NPR2 is involved in FSH-mediated mouse oocyte meiotic resumption. J Ovarian Res. 2016;9:6. 26880031 10.1186/s13048-016-0218-y PMC4754804

[41] Liu CL Cheng L Ko CH. Bioassay-guided isolation of anti-inflammatory components from the root of Rehmannia glutinosa and its underlying mechanism via inhibition of iNOS pathway. J Ethnopharmacol. 2012;143:867–75. 23034094 10.1016/j.jep.2012.08.012

[42] Ramasamy R Yan SF Schmidt AM. Receptor for AGE (RAGE): signaling mechanisms in the pathogenesis of diabetes and its complications. Ann N Y Acad Sci. 2011;1243:88–102. 22211895 10.1111/j.1749-6632.2011.06320.x PMC4501013

[43] Merhi Z Buyuk E Cipolla MJ. Advanced glycation end products alter steroidogenic gene expression by granulosa cells: an effect partially reversible by vitamin D. Mol Hum Reprod. 2018;24:318–26. 29538679 10.1093/molehr/gay014 PMC6530817

